# L-Theanine Administration Modulates the Absorption of Dietary Nutrients and Expression of Transporters and Receptors in the Intestinal Mucosa of Rats

**DOI:** 10.1155/2017/9747256

**Published:** 2017-07-24

**Authors:** Qiongxian Yan, Haiou Tong, Shaoxun Tang, Zhiliang Tan, Xuefeng Han, Chuanshe Zhou

**Affiliations:** ^1^Key Laboratory of Agro-Ecological Processes in Subtropical Region, Hunan Research Center of Livestock & Poultry Sciences, South-Central Experimental Station of Animal Nutrition and Feed Science in Ministry of Agriculture, Institute of Subtropical Agriculture, The Chinese Academy of Sciences, Changsha, Hunan 410125, China; ^2^Hunan Co-Innovation Center for Utilization of Botanical Functional Ingredients, Changsha 410128, China; ^3^Hunan Co-Innovation Center of Animal Production Safety, CICAPS, Changsha, Hunan 410128, China

## Abstract

L-theanine has various advantageous functions for human health; whether or not it could mediate the nutrients absorption is unknown yet. The effects of L-theanine on intestinal nutrients absorption were investigated using rats ingesting L-theanine solution (0, 50, 200, and 400 mg/kg body weight) per day for two weeks. The decline of insulin secretion and glucose concentration in the serum was observed by L-theanine. Urea and high-density lipoprotein were also reduced by 50 mg/kg L-theanine. Jejunal and ileac basic amino acids transporters* SLC7a1* and* SLC7a9*, neutral* SLC1a5* and* SLC16a10*, and acidic* SLC1a1* expression were upregulated. The expression of intestinal* SGLT3* and* GLUT5* responsible for carbohydrates uptake and* GPR120* and* FABP2* associated with fatty acids transport were inhibited. These results indicated that L-theanine could inhibit the glucose uptake by downregulating the related gene expression in the small intestine of rats. Intestinal gene expression of transporters responding to amino acids absorption was stimulated by L-theanine administration.

## 1. Introduction

L-theanine, as a non-protein-forming amino acid (AA), contributes to the umami taste and unique flavor of green tea. Its content in tea leaves is closely related to the quality and price of green tea [1, 2]. L-theanine is beneficial for remedying various nutritional and metabolic diseases in human, including providing antiobesity effects [3, 4], suppressing the body weight increases and fat accumulation [3, 5], and exerting antidiabetic effects [6, 7]. L-theanine is transported through the intestinal brush border membrane mainly via neutral AA systems B, A, ASC, N, and L, based on findings that L-theanine inhibited the absorption of glutamine and large neutral amino acids (AAs, leucine, and tryptophan) into organs [8–10]. Our knowledge data and previous findings also confirmed that most neutral AAs (threonine, valine, methionine, isoleucine, serine, alanine, tyrosine, and leucine) and certain basic AA (lysine) in the serum of L-theanine-administered rats were decreased [8, 11]. These researches indicated that L-theanine could competitively suppress the absorption of AAs.

However, AAs absorption is dependent on the activities of AA transporters located in the brush border membrane of small intestine. Neutral AA transporters, solute carrier family 1, member 5* (SLC1a5)* and family 16, member 10* (SLC16a10)*, are responsible for threonine, serine, alanine, cysteine, glutamine and phenylalanine, tyrosine, and tryptophan transporting, respectively. Basic AA transporters, solute carrier family 7, member 1* (SLC7a1)* and member 9* (SLC7a9)*, are in charge of transporting arginine, lysine, histidine, alanine, serine, cysteine, threonine, asparagine, and glutamine. Acidic AA transporters solute carrier family 1, member 1* (SLC1a1)* and member 2* (SLC1a2)* transport glutamate and aspartate. It is reported that L-theanine competitively inhibited the uptake of glutamate substrate through solute carrier family 1, member 3* (SLC1a3)* and* SLC1a2 *expressed in cancer cells [12, 13]. However, the expression pattern of glutamate transporter subtypes in tumor cells is different from normal cells. Therefore, it is necessary to investigate the efficacy of L-theanine on glutamate transporters in normal tissues. Whether or not the expression of different AA transport systems is mediated by L-theanine is unknown yet.

Furthermore, it is reported that the fatty accumulation in mice was suppressed by the administration of green tea powder [4] and theanine was responsible for this suppressive effect [3]. Although serum glucose in rats was not changed, the insulin was reduced by oral theanine [14]. These literatures indicate that metabolism of lipid and insulin is regulated by L-theanine. In the enterocytes of rats, there are many transporters and receptors responses to sugar and fatty acids transport, including sodium dependent glucose transporters* (SGLTs)*, glucose transporters, G-protein-coupled receptors, and fatty acid binding protein 2* (FABP2)* [15–21]. Whether these transporters and receptors involved in the regulation of L-theanine administration on absorption of glucose and lipid is unclear. Based on these questions, we measured the nutrient content in the blood and mRNA expression of related transporters and receptors in small intestine of rats after the intragastric administration of L-theanine for two weeks, aiming at figuring out the preliminary L-theanine-induced regulation mechanism in nutrients absorption in rats.

## 2. Material and Methods

### 2.1. Experimental Design

This experiment was conducted according to the animal care guidelines of the Animal Care Committee, Institute of Subtropical Agriculture, the Chinese Academy of Sciences, Changsha city, Hunan province, China (number KYNEAAM-2013-0009). Sixty-four Sprague Dawley (SD) rats which are 3 weeks old weighing 74–92.2 g were used as experimental animals. The management of SD rats and L-theanine administration experiment was the same as Li et al. [22]. The animals were individually housed in plastic cages under laboratory conditions (25 ± 3°C, 70 ± 5% relative humidity, good ventilation, and a 12-h light-dark cycle) and had free access to food and pure water. After three days of adaptation, SD rats were randomly divided into four treatment groups. Each group contained eight male rats and eight female rats. During fasting (15:00–17:00 h), rats in the treatments received gastric intubation of four different doses of L-theanine (0, 50, 200, and 400 mg/kg body weight/day), respectively. L-theanine was freshly dissolved in 0.9% NaCl solution in advance before intubation every day. 1 mL of the L-theanine solution was daily administered to each rat for two weeks.

### 2.2. Blood and Tissue Samples Collection

At the end of the experiment, SD rats were fasted overnight and anesthetized by ether for 4 min, and then blood was collected from the jugular vein into tubes without anticoagulant. The blood samples were centrifuged at 3500 rpm for 15 min at 4°C, and then serum samples were collected and stored at −80°C until assay. The whole jejunum and ileum segments were collected and rinsed with ice-cold saline (0.9% NaCl wt/vol). Then the mucosae were carefully removed, quickly frozen in liquid nitrogen, and stored at −80°C prior to subsequent analyses.

### 2.3. Analysis of Serum

The glucose, total cholesterol, triglyceride (TG), urea, low-density lipoprotein cholesterol (LDL), and high-density lipoprotein cholesterol (HDL) were determined by automatic biochemistry analyzer (Synchron Clinical System CX4 PRO, Beckman Coulter, USA) according to the instructions. Insulin was assayed by the ELISA kit purchased from Huamei Biotechnology Co., Ltd. (Wuhan, Hubei, China). Non-esterified fatty acids (NEFA) were measured by kit produced by Nanjing Jiancheng Bioengineering Institute (Nanjing, Jiangsu, China).

### 2.4. Real-Time Quantitative PCR

Total RNA was isolated from the mucosa of jejunum and ileum using the Trizol Reagent (Invitrogen, USA), and* c*DNA was synthesized using the Revert Aid First Strand* c*DNA synthesis kit (Applied Biosystems, Thermo Fisher Scientific, USA). For relative quantification of gene expression, the ABI Prism 7900 HT Fast Real-Time PCR System (Applied Biosystems, Foster, CA) was used. Primers were designed using the Primer 3 plus program, and sequences are listed in Table 1. The reaction system contained 5 *μ*L SYBR® Premix Ex Taq™ (2x), 0.4 *μ*L PCR forward primer (10 *μ*M), 0.4 *μ*L PCR reverse primer (10 *μ*M), 0.2 *μ*L ROX reference dye (50x), 1.0 *μ*L* c*DNA, and 3 *μ*L sterilized ddH_2_O. The thermal profile for all reactions was 30 s at 95°C, then 40 cycles of denaturation at 95°C for 5 s, and annealing at 60°C for 30 s. Each reaction was completed with a melting curve analysis to ensure the specificity of the reaction. All the samples were analyzed in duplicate, and the relative amount of each specific transcript was obtained after normalization against the endogenous control *β*-actin. The relative amounts of target genes were quantified according to the 2^−ΔΔCT^ method [23].

### 2.5. Statistical Analysis

Statistical analyses were conducted by one-way analysis of variance (ANOVA) using the Mixed Proc of SAS (version 8.2, SAS Institute, Cary, NC, USA). The main effect tested was the dose of L-theanine. When indicated by ANOVA, means were separated using least significant differences. Significance was declared at* P* < 0.05.

## 3. Results

As shown in Table 2, glucose concentration was decreased by 400 mg/kg L-theanine administration compared to the control group (0 mg/kg L-theanine administration) (*P *< 0.05). Insulin concentration was linearly decreased by L-theanine administration (*P *< 0.001). There were no differences (*P *> 0.05) in the serum cholesterol, TG, NEFA, and LDL concentrations among the L-theanine treatments. Concentrations of urea and HDL were decreased by 50 mg/kg L-theanine treatment compared to the control group (*P *< 0.05).

Transcript levels of intestinal AA transporters in the intestine of rats are shown in Table 3. Expression of acidic AA transporter* SLC1a1* was upregulated in the jejunum and ileum (Quadratic,* P *< 0.001), while jejunal* SLC1a2* transcript was linearly decreased (*P *< 0.001) with the increasing doses of L-theanine but increased by L-theanine treatments in the ileum (Quadratic,* P *< 0.05). Expression of neutral AA transporter* SLC1a5* was increased by doses of L-theanine (jejunum, Linear,* P *< 0.001; ileum, Linear and Quadratic,* P *< 0.001); therein the maximal values both occurred in the 400 mg/kg L-theanine treatment. Another neutral AA transporter* SLC16a10 *expression in the jejunum and ileum was upregulated by doses of L-theanine (Quadratic,* P *< 0.001). Basic AA transporters* SLC7a1* (jejunum, Linear,* P *< 0.01; ileum, Linear and Quadratic,* P *< 0.001) and* SLC7a9 *expression (jejunum, Quadratic,* P *< 0.001; ileum, Linear and Quadratic,* P *< 0.001) was increased with the increasing doses of L-theanine.

Gene expressions of glucose transporters and receptors in the intestine of rats are shown in Table 4. Transcript level of* SGLT1* in the jejunum was stimulated (*P *< 0.05) by 400 mg/kg L-theanine compared to the 200 mg/kg L-theanine treatment, while in the ileum it was downregulated (Quadratic,* P *< 0.001); therein a minimum value appeared at the 200 mg/kg L-theanine group.* SGLT3 *expression in the jejunum and ileum was decreased by L-theanine treatment (jejunum, Linear,* P *< 0.001; ileum, Linear and Quadratic,* P *< 0.001). Comparing with the 50 mg/kg L-theanine treatment, jejunal* GLUT2* expression was suppressed (*P *< 0.05) by 200 mg/kg L-theanine. Ileac* GLUT2* expression was upregulated (*P *< 0.01) by 50 mg/kg L-theanine and then inhibited (*P *< 0.05) by high doses of L-theanine treatments compared to 50 mg/kg L-theanine. Jejunal* GLUT5* expression was inhibited (*P *< 0.05) by high doses of L-theanine treatment compared to the control group, while its expression in the ileum was linearly decreased (*P *< 0.01) by increasing doses of L-theanine.

The mRNA abundance of the fatty acid transporters and receptors in the intestine of rats is shown in Table 5. Jejunal* FATP* expression was decreased by L-theanine treatments (Quadratic,* P *< 0.01), while its expression levels in the treatments of 50 and 400 mg/kg L-theanine were lower (*P *< 0.01) than that of control group and 200 mg/kg L-theanine treatment. Ileac* FATP* expression was not affected by L-theanine treatments (*P *> 0.05). Jejunal* GPR43* expression was unchanged by L-theanine treatments (*P *> 0.05). However, its expression in the ileum of 50 mg/kg L-theanine treatment was decreased (*P *< 0.05) compared with control group and 200 mg/kg L-theanine treatment.* GPR120* (jejunum, Linear and Quadratic,* P *< 0.001; ileum, Linear,* P *< 0.001) and* FABP2 *(jejunum, Linear,* P *< 0.001; ileum, Linear and Quadratic,* P *< 0.001) expression levels were both suppressed by L-theanine treatments.

## 4. Discussion

To the best of our knowledge, this experiment is a new attempt to investigate the link between serum nutrients and the expression of nutrient-associated transporters and receptors in the small intestine of L-theanine-administered rats. In this study, the declines of glucose, insulin, and urea in the serum were observed by L-theanine administration, indicating that L-theanine could inhibit the absorption of glucose, nitrogen, and secretion of insulin. Our results are partly in line with the data of Yamada et al. (2008) which observed reduced insulin level with unchanged glucose concentration in the serum of rats administrated by 4 g/kg oral L-theanine. These results are inconsistent with the findings of Zheng et al. (2004) which discovered that TG and NEFA levels in the serum of mice were decreased by 0.03% L-theanine administration. This discrepancy appears to be due to the dosage of L-theanine ingested, method of administration, and experimental period.

The upregulating effects of L-theanine are reflected in the AA transporters expression at the mRNA level in small intestine in this study, except* SLC1a2*. This finding can partly explain the increased AAs concentrations in rat serum after L-theanine ingestion [11], including acidic acid (aspartic acid and glutamic acid), neutral acid (glutamine), and basic acid (histidine) (see Supplemental Table 1 in [11]; see Supplementary Material available online at https://doi.org/10.1155/2017/9747256), indicating that L-theanine promotes the AAs absorption in rat small intestine. The opposing effect of L-theanine on jejunal* SLC1a2* expression was observed, reflecting that asparagine absorption in the jejunum might be blocked by L-theanine. Although direct evidences about the regulatory mechanism of AA transporters transcription by L-theanine are lacking, previous literatures showed that activating transcription factor 4 (ATF4) could transcriptionally upregulate* SLC7a1* [24] and regulatory factor X proteins* (RFXs)* induced mRNA of* SLC1a1* [25]. After MatInspector online analysis [26], we find that there are* ATF4* binding sites in the promoter regions of* SLC7a1* (between nucleotides +10 and +18) and* SLC7a9* (between nucleotides −155 and −146) genes and* RXFs* located in* SLC1a1 *(between nucleotides −239 and −86) promoter sequence. Additionally, elements for E-box binding factors (*EBOX*) and cAMP-responsive element binding proteins* (CREB)* binding are identified in the promoter sequences of* SLC1a5*,* SLC7a1*, and* SLC7a9 *genes. Therefore, we speculated that L-theanine, as an amino acid, changed* SLC1a1*,* SLC1a5*,* SLC7a1*, and* SLC7a9 *mRNA transcription via acting with* ATF4*,* RXF*,* EBOX*, and* CREB* proteins.

Glucose transporting from the intestinal lumen to the blood mainly depends on Na^+^-glucose cotransporter* SGLT1*, which absorbs glucose and galactose and the passive glucose transporter* GLUT2*, which acts as a glucose sensor [27–30].* SGLT3* is also a glucose sensor in cholinergic neurons neighboring enterocytes and induces membrane currents upon Na^+^-glucose binding [27].* GLUT5* is primarily in charge of fructose absorption into the cytosol. Although decreases in* SGLT1 *and* GLUT2* mRNA abundance in the intestine of rats receiving 200 mg/kg L-theanine, in which glucose absorption was declined, were not observed in this study, we found that intestinal* SGLT3* and ileac* GLUT5* transcripts in L-theanine-ingested rats were decreased in a dose-dependent manner. These results indicated that rats intestinal* GLUT2* was less impressible than* GLUT5* to L-theanine administration at the transcriptional level, and* SGLT3* and* GLUT5 *genes rather than* SGLT1 *and* GLUT2 *play a role in intestinal glucose absorption of L-theanine-ingested rats. It is reported that period circadian clock 1* (PER1)* exerted an indirect suppressive effect on rat* SGLT1* promoter [31] and hepatic nuclear factors* (HNF)* regulated* SGLT1* and* GLUT2* promoter activities [32, 33]. By analyzing [26], we also identified* HNF*-element located in* SGLT3 *and peroxisome proliferator-activated receptor* (PPARG)* element encompassed in* GLUT5* promoter regions. Therefore, we predicted that L-theanine may target transcription factors (*PER1*,* HNF*, and* PPARG*) and further inhibit the expression of glucose transporters mRNA.

It is reported that* GPR43* binds short-chain fatty acids, whereas* GPR120* responds to medium and long chain fatty acids [34, 35].* FABP2* also displays high-affinity binding for long chain fatty acids and is believed to be involved with uptake and trafficking of lipids in the intestine [21]. In the present study,* GPR120* and* FABP2* transcripts in jejunum and ileum were decreased by L-theanine. Jejunal* FATP* mRNA was also suppressed by 50 mg/kg and 400 mg/kg L-theanine. However, triglyceride and cholesterol contents in the serum of L-theanine-treated rats were not affected (Table 2). These results state that the intestinal uptake of dietary fatty acids might have been inhibited by L-theanine. Further research is needed to explore the regulatory mechanism of L-theanine on intestinal uptake of dietary lipids.

In summary, L-theanine administration had decreased serum glucose probably by inhibiting intestinal* SGLT3 *and* GLUT5* mRNA expression in rats. Dietary fatty acids uptake might be suppressed by downregulating* GPR120* and* FABP2* transcripts in the intestine of rats. Meanwhile, intestinal transporters responding to AAs absorption were upregulated by L-theanine administration. Our data provide theoretical basis for further investigation of L-theanine and nutrients interaction.

## Supplementary Material

Effects of L-Theanine administration on serum amino acids profiles in rat.

## Figures and Tables

**Table 1:** Sequences of primers used for real-time quantitative PCR.

Gene	GenBank accession	Primer	Length (bp)
*SLC7a1*	NM_013111.3	Forward-CCTTCATCACTGGCTGGAAC Reverse-GGTTTGCCTATCAGCTCGTC	100
*SLC1a5*	NM_175758.3	Forward-GGAGAAATGGACTGGGTGTG Reverse-CCAGCAAGAAGGCTCTGAAT	107
*SLC1a2*	NM_001035233.1	Forward-GGCAGTCATCTCCCTGTTGA Reverse-AGACATTCATCCCGTCCTTG	101
*SLC1a1*	NM_013032.3	Forward-GGAGTCTTGGTTCGAGGACA Reverse-GTGGCAGAATGACGAGCTTC	106
*SLC16a10*	NM_138831.1	Forward-TCACTGGTCATTCTGGGACA Reverse-CCTAACAGCAAAGGGAGCAA	107
*SLC7a9 *	NM_053929.1	Forward-ACCAAGTCAGGGGGTGAGTA Reverse-AGATGATGGCGAAGGATGAG	115
*SGLT1*	NM_013033	Forward-GCCATCATCCTCTTCGCTAT Reverse-CGCTCTTCTGTGCTGTTACG	122
*SGLT3*	NM_001106383	Forward-GATGCTGGTGCTGAAACTGA Reverse-CGCTGTTGAAGATGGAGGTC	101
*GLUT2*	NM_012879	Forward-GATTGCTCCAACCACACTCA Reverse-CCTGATTGCCCAGAATGAAG	113
*GLUT5*	NM_031741	Forward-GGGCTCTTGGTCACACACA Reverse-CGTCTTGTCTCTCGGCAACT	108
*FATP*	NM_053580.2	Forward-GGAAGGTTGCTGTGGTGTTC Reverse-ATGGGAGCCAGAAGGGTAGA	120
*GPR43*	NM_001005877	Forward-AGGCTGTGGTGTTCAGTTCC Reverse-GGGATTGCGGAGTAGTAGCA	113
*GPR120*	NM_001047088.1	Forward-AGACCACCGTTCTGGGACT Reverse-GAAGAGGTTGAGCACCAAGC	119
*FABP2*	NM_013068.1	Forward-GAGGCCAAGCGGATCTTTA Reverse-TGCATTATCAGCGAGATGGA	109
*β-actin *	NM_031144.3	Forward-TGTCACCAACTGGGACGATA Reverse-GGGGTGTTGAAGGTCTCAAA	165

**Table 2:** Effects of L-theanine administration on average daily gain and biochemical parameters in serum of rats.

Item	Treatments (mg/kg BW·d)				*P* value	
	0	50	200	400	Linear	Quadratic
Average daily gain, g/d	5.24 ± 0.17^b^	6.01 ± 0.17^a^	6.15 ± 0.17^a^	5.98 ± 0.17^a^	0.038	<0.01
Glucose, mM	5.65 ± 0.29^a^	5.31 ± 0.29^ab^	5.74 ± 0.29^a^	4.77 ± 0.30^b^	NS	NS
Insulin, uIU/mL	43.2 ± 2.18^a^	41.7 ± 2.18^a^	26.3 ± 2.26^b^	19.0 ± 3.08^c^	<0.001	NS
Cholesterol, mM	2.23 ± 0.09	2.08 ± 0.09	2.13 ± 0.09	2.05 ± 0.09	NS	NS
Triglyceride, mM	1.23 ± 0.07	1.21 ± 0.07	1.02 ± 0.07	1.16 ± 0.07	NS	NS
NEFA, mM	1.27 ± 0.13	1.35 ± 0.10	1.45 ± 0.11	1.31 ± 0.10	NS	NS
Urea, mM	5.42 ± 0.16^a^	4.85 ± 0.16^b^	5.70 ± 0.16^a^	5.38 ± 0.17^a^	NS	NS
LDL, mM	0.339 ± 0.02	0.341 ± 0.02	0.354 ± 0.02	0.347 ± 0.02	NS	NS
HDL, mM	1.74 ± 0.06^a^	1.52 ± 0.06^b^	1.60 ± 0.06^ab^	1.60 ± 0.06^ab^	NS	NS

BW: body weight, NEFA: non-esterified fatty acids, LDL: low-density lipoprotein, HDL: high-density lipoprotein, and NS: not significant. ^a–c^ Means within a row not bearing a common superscript letter differ (*P* < 0.05). Data were reported as mean ± SE. Data of average daily gain were cited by Tong et al. (2016).

**Table 3:** Effects of L-theanine administration on the transcript levels of AA transporters in rat intestine.

Item	Treatments (mg/kg BW·d)				*P* value	
	0	50	200	400	Linear	Quadratic
Jejunum						
* SLC1a1*	1.00^b^	1.71 ± 0.43^b^	7.70 ± 0.50^a^	1.12 ± 0.55^b^	NS	<0.001
* SLC1a2*	1.00^a^	0.34 ± 0.06^c^	0.63 ± 0.07^b^	0.23 ± 0.07^c^	<0.001	NS
* SLC1a5*	1.00^b^	1.77 ± 0.43^b^	1.61 ± 0.38^b^	3.49 ± 0.41^a^	<0.001	NS
* SLC16a10*	1.00^c^	7.17 ± 0.84^a^	5.25 ± 0.79^ab^	3.83 ± 0.68^b^	NS	<0.001
* SLC7a1*	1.00^c^	8.40 ± 0.70^a^	3.93 ± 0.90^b^	8.88 ± 1.28^a^	0.0017	NS
* SLC7a9*	1.00^c^	6.40 ± 0.60^b^	10.9 ± 0.66^a^	1.70 ± 0.60^c^	NS	<0.001
Ileum						
* SLC1a1*	1.00^b^	4.89 ± 0.35^a^	5.11 ± 0.39^a^	1.25 ± 0.39^b^	NS	<0.001
* SLC1a2*	1.00	1.47 ± 0.29	1.86 ± 0.24	1.02 ± 0.44	NS	0.01
* SLC1a5*	1.00^b^	1.45 ± 0.35^b^	0.94 ± 0.37^b^	4.45 ± 0.45^a^	<0.001	<0.001
* SLC16a10*	1.00^c^	8.27 ± 0.27^b^	13.1 ± 0.63^a^	1.52 ± 0.77^c^	NS	<0.001
* SLC7a1*	1.00^b^	1.71 ± 0.34^b^	1.18 ± 0.36^b^	4.62 ± 0.45^a^	<0.001	<0.001
* SLC7a9*	1.00^b^	1.93 ± 0.42^b^	7.21 ± 0.52^a^	2.09 ± 0.42^b^	<0.001	<0.001

BW: body weight, *SLC1a1*: solute carrier family 1, member 1, *SLC1a2*: solute carrier family 1, member 2, *SLC1a5*: solute carrier family 1, member 5, *SLC16a10*: solute carrier family 16, member 10, *SLC7a1*: solute carrier family 7, member 1, *SLC7a9*: solute carrier family 7, member 9, and NS: not significant.  ^a–c^Means within a row not bearing a common superscript letter differ (*P* < 0.05). Data were reported as mean ± SE.

**Table 4:** Effects of L-theanine administration on the transcript levels of glucose transporters and receptors in rat intestine.

Item	Treatments (mg/kg BW·d)				*P* value	
	0	50	200	400	Linear	Quadratic
Jejunum						
* SGLT1*	1.00^ab^	1.14 ± 0.20^ab^	0.80 ± 0.21^b^	1.65 ± 0.21^a^	NS	NS
* SGLT3*	1.00^ab^	1.06 ± 0.15^a^	0.53 ± 0.16^b^	0.03 ± 0.15^c^	<0.001	NS
* GLUT2*	1.00^ab^	1.44 ± 0.2^a^	0.77 ± 0.20^b^	1.12 ± 0.17^ab^	NS	NS
* GLUT5*	1.00^a^	0.16 ± 0.08^c^	0.72 ± 0.10^b^	0.68 ± 0.10^b^	NS	NS
Ileum						
* SGLT1*	1.00^a^	0.60 ± 0.12^b^	0.17 ± 0.11^c^	1.10 ± 0.09^a^	NS	<0.001
* SGLT3 *	1.00^a^	0.61 ± 0.07^b^	0.26 ± 0.06^c^	0.01 ± 0.005^d^	<0.001	<0.001
* GLUT2*	1.00^c^	3.07 ± 0.30^a^	1.31 ± 0.33^bc^	1.99 ± 0.30^b^	NS	NS
* GLUT5*	1.00^a^	0.58 ± 0.10^b^	0.53 ± 0.11^b^	0.46 ± 0.11^b^	0.003	NS

BW: body weight, *SGLT1*: sodium dependent glucose transporter 1, *SGLT3*: sodium dependent glucose transporter 3, *GLUT2*: glucose transporter protein, member 2, *GLUT5*: glucose transporter protein, member 5, and NS: not significant.  ^a–d^Means within a row not bearing a common superscript letter differ (*P* < 0.05). Data were reported as mean ± SE.

**Table 5:** Effects of L-theanine administration on the transcript levels of fatty acid transporters and receptors in rat intestine.

Item	Treatments (mg/kg BW·d)				*P* value	
	0	50	200	400	Linear	Quadratic
Jejunum						
* FATP*	1.00^ab^	0.27 ± 0.03^b^	1.86 ± 0.07^a^	0.40 ± 0.03^b^	NS	0.007
* GPR43*	1.00	0.79 ± 0.49	1.23 ± 0.57	0.86 ± 0.61	NS	NS
* GPR120*	1.00^a^	0.25 ± 0.03^b^	0.18 ± 0.03^b^	0.06 ± 0.03^c^	<0.001	<0.001
* FABP2*	1.00^a^	0.52 ± 0.08^b^	0.51 ± 0.08^b^	0.09 ± 0.08^c^	<0.001	NS
Ileum						
* FATP*	1.00	1.52 ± 0.19	1.00 ± 0.19	1.76 ± 0.39	NS	NS
* GPR43*	1.00^a^	0.56 ± 0.13^b^	0.72 ± 0.14^ab^	1.02 ± 0.14^a^	NS	0.07
* GPR120*	1.00^a^	0.36 ± 0.11^bc^	0.64 ± 0.11^b^	0.21 ± 0.12^c^	<0.001	NS
* FABP2*	1.00^a^	0.38 ± 0.05^b^	0.16 ± 0.05^c^	0.08 ± 0.05^c^	<0.001	<0.001

BW: body weight, FATP: fatty acid transport protein, GPR43: G-protein-coupled receptor 43, GPR120: G-protein-coupled receptor 120, FABP2: fatty acid binding protein 2, and NS: not significant. ^a–c^Means within a row not bearing a common superscript letter differ (*P* < 0.05). Data were reported as mean ± SE.

## Abbreviations

AA: Amino acid
*ATF4*: Activating transcription factor 4
cDNA: Complementary DNA
*CREB*: cAMP-responsive element binding proteins
ddH_2_O: Distilled water
*EBOX*: E-box binding factors
ELISA: Enzyme-linked immunosorbent assay
*FABP2*: Fatty acid binding protein 2
*FATP*: Fatty acid transport protein
*GLUT2*: Glucose transporter protein, member 2
*GLUT5*: Glucose transporter protein, member 5
*GPR43*: G-protein-coupled receptor 43
*GPR120*: G-protein-coupled receptor 120
HDL: High-density lipoprotein cholesterol
HNF: Hepatic nuclear factors
LDL: Low-density lipoprotein cholesterol
mRNA: Messenger RNA
NEFA: Non-esterified fatty acids
*PER1*: Period circadian clock 1
*PPARG*: Peroxisome proliferator-activated receptor
*RFXs*: Regulatory factor X proteins
*SLC1a1*: Solute carrier family 1, member 1
*SLC1a2*: Solute carrier family 1, member 2
*SLC1a5*: Solute carrier family 1, member 5
*SLC16a10*: Solute carrier family 16, member 10
*SLC7a1*: Solute carrier family 7, member 1
*SLC7a9*: Solute carrier family 7, member 9
*SGLT1*: Sodium dependent glucose transporter 1
*SGLT3*: Sodium dependent glucose transporter 3
SD: Sprague Dawley
TG: Triglyceride.

## References

[1] Chu D. C., Yamamoto T., Juneja L. R., Chu D. C., Kim M. (1997). Green tea-its cultivation, processing of the leaves for drinking materials, and kinds of green tea. *Chemistry and Applications of Green Tea*.

[2] Vuong Q. V., Bowyer M. C., Roach P. D. (2011). L-Theanine: Properties, synthesis and isolation from tea. *Journal of the Science of Food and Agriculture*.

[3] Zheng G., Sayama K., Okubo T., Juneja L. R., Oguni I. (2004). Anti-obesity effects of three major components of green tea, catechins, caffeine and theanine, in mice. *In Vivo*.

[4] Sayama K., Lin S., Zheng G., Oguni I. (2000). Effects of green tea on growth, food utilization and lipid metabolism in mice. *In Vivo*.

[5] Takagi Y., Kurihara S., Higashi N. (2010). Combined administration of L-cystine and L-theanine enhances immune functions and protects against influenza virus infection in aged mice. *Journal of Veterinary Medical Science*.

[6] Matsumoto K., Yamamoto S., Yoshikawa Y. (2005). Antidiabetic activity of Zn(II) complexes with a derivative of L-glutamine. *Bulletin of the Chemical Society of Japan*.

[7] Kajiwara N., Yoshikawa Y., Yasui H., Matsumoto K. (2013). Experimental observations of anti-diabetic activity of zinc complexes with theanine. *Annals of Nutrition and Metabolism*.

[8] Terashima T., Takido J., Yokogoshi H. (1999). Time-dependent changes of amino acids in the serum, liver, brain and urine of rats administered with theanine. *Bioscience, Biotechnology and Biochemistry*.

[9] Kitaoka S., Hayashi H., Yokogoshi H., Suzuki Y. (1996). Transmural potential changes associated with the in vitro absorption of theanine in the guinea pig intestine. *Bioscience, Biotechnology and Biochemistry*.

[10] Kakuda T., Hinoi E., Abe A., Nozawa A., Ogura M., Yoneda Y. (2008). Theanine, an ingredient of green tea, inhibits [3H] glutamine transport in neurons and astroglia in rat brain. *Journal of Neuroscience Research*.

[11] Tong H. O., Li C. J., Yan Q. X., Tan Z. L., Han X. F. (2016). Effects of *L*-theanine on serum and liver amino acid compositions in weaning rats. *Food science*.

[12] Sugiyama T., Sadzuka Y., Tanaka K., Sonobe T. (2001). Inhibition of glutamate transporter by theanine enhances the therapeutic efficacy of doxorubicin. *Toxicology Letters*.

[13] Sugiyama T., Sadzuka Y. (2003). Theanine and glutamate transporter inhibitors enhance the antitumor efficacy of chemotherapeutic agents. *Biochimica et Biophysica Acta - Reviews on Cancer*.

[14] Yamada T., Nishimura Y., Sakurai T. (2008). Administration of theanine, a unique amino acid in tea leaves, changed feeding-relating components in serum and feeding behavior in rats. *Bioscience, Biotechnology and Biochemistry*.

[15] Veyhl M., Spangenberg J., Püschel B. (1993). Cloning of a membrane-associated protein which modifies activity and properties of the Na+-D-glucose cotransporter. *Journal of Biological Chemistry*.

[16] Kellett G. L., Helliwell P. A. (2000). The diffusive component of intestinal glucose absorption is mediated by the glucose-induced recruitment of GLUT2 to the brush-border membrane. *Biochemical Journal*.

[17] Tazawa S., Yamato T., Fujikura H. (2005). SLC5A9/SGLT4, a new Na+-dependent glucose transporter, is an essential transporter for mannose, 1, 5-anhydro-D-glucitol, and fructose. *Life Sciences*.

[18] Karaki S., Mitsui R., Hayashi H. (2006). Short-chain fatty acid receptor, GPR43, is expressed by enteroendocrine cells and mucosal mast cells in rat intestine. *Cell and Tissue Research*.

[19] Kaji I., Karaki S.-I., Tanaka R., Kuwahara A. (2011). Density distribution of free fatty acid receptor 2 (FFA2)-expressing and GLP-1-producing enteroendocrine L cells in human and rat lower intestine, and increased cell numbers after ingestion of fructo-oligosaccharide. *Journal of Molecular Histology*.

[20] Paulsen S. J., Larsen L. K., Hansen G., Chelur S., Larsen P. J., Vrang N. (2014). Expression of the fatty acid receptor GPR120 in the gut of diet-induced-obese rats and its role in GLP-1 secretion. *PLoS ONE*.

[21] Gajda A. M., Storch J. (2015). Enterocyte fatty acid-binding proteins (FABPs): different functions of liver and intestinal FABPs in the intestine. *Prostaglandins Leukotrienes and Essential Fatty Acids*.

[22] Li C., Tong H., Yan Q. (2016). L-theanine improves immunity by altering th2/th1 cytokine balance, brain neurotransmitters, and expression of phospholipase c in rat hearts. *Medical Science Monitor*.

[23] Livak K. J., Schmittgen T. D. (2001). Analysis of relative gene expression data using real-time quantitative PCR and the 2(-Delta Delta C(T)) method. *Methods*.

[24] Adams C. M. (2007). Role of the transcription factor ATF4 in the anabolic actions of insulin and the anti-anabolic actions of glucocorticoids. *Journal of Biological Chemistry*.

[25] Ma K., Zheng S., Zuo Z. (2006). The transcription factor regulatory factor X1 increases the expression of neuronal glutamate transporter type 3. *Journal of Biological Chemistry*.

[26] Cartharius K., Frech K., Grote K. (2005). MatInspector and beyond: promoter analysis based on transcription factor binding sites. *Bioinformatics*.

[27] Díez-Sampedro A., Hirayama B. A., Osswald C. (2003). A glucose sensor hiding in a family of transporters. *Proceedings of the National Academy of Sciences of the United States of America*.

[28] Wright E. M., Loo D. D. F., Hirayama B. A., Turk E. (2004). Surprising versatility of Na+-glucose cotransporters: SLC5. *Physiology*.

[29] Hediger M. A., Turk E., Wright E. M. (1989). Homology of the human intestinal Na+/glucose and Escherichia coli Na+/proline cotransporters. *Proceedings of the National Academy of Sciences of the United States of America*.

[30] Hediger M. A., Coady M. J., Ikeda T. S., Wright E. M. (1987). Expression cloning and cDNA sequencing of the Na+/glucose co-transporter. *Nature*.

[31] Balakrishnan A., Stearns A. T., Ashley S. W., Rhoads D. B., Tavakkolizadeh A. (2012). PER1 modulates SGLT1 transcription in vitro independent of E-box status. *Digestive Diseases and Sciences*.

[32] Rhoads D. B., Rosenbaum D. H., Unsal H., Isselbacher K. J., Levitsky L. L. (1998). Circadian periodicity of intestinal Na+/glucose cotransporter 1 mRNA levels is transcriptionally regulated. *Journal of Biological Chemistry*.

[33] Kekuda R., Saha P., Sundaram U. (2008). Role of Sp1 and HNF1 transcription factors in SGLT1 regulation during chronic intestinal inflammation. *American Journal of Physiology—Gastrointestinal and Liver Physiology*.

[34] Nilsson N. E., Kotarsky K., Owman C., Olde B. (2003). Identification of a free fatty acid receptor, FFA2R, expressed on leukocytes and activated by short-chain fatty acids. *Biochemical and Biophysical Research Communications*.

[35] Brown A. J., Goldsworthy S. M., Barnes A. A. (2003). The orphan G protein-coupled receptors GPR41 and GPR43 are activated by propionate and other short chain carboxylic acids. *Journal of Biological Chemistry*.

