# Supplementary material for: L-Theanine Administration Modulates the Absorption of Dietary Nutrients and Expression of Transporters and Receptors in the Intestinal Mucosa of Rats
Source: Biomed Res Int. 2017 Jul 24;2017:9747256. doi: 10.1155/2017/9747256 (PMC5546063; doi:10.1155/2017/9747256)
Supplement: Supplementary file 1 — Effects of L-Theanine administration on serum amino acids profiles in rat. [file 9747256.f1.docx]

**TABLE1: Effects of L-Theanine on serum amino acids profiles (μmol/L).**

| Item^1^ | Treatments(mg/kg BW·d) | | | |  | *P* value^2^ | |
| --- | --- | --- | --- | --- | --- | --- | --- |
|  | 0 | 50 | 200 | 400 |  | Linear | Quadratic |
| Thr | 148.29 ±10.89^b^ | 179.83±10.25^a^ | 165.78±9.61^ab^ | 107.78±11.37^c^ |  | ** | ** |
| Val | 112.28 ±8.13^a^ | 129.01±7.66^a^ | 114.27±7.18^a^ | 73.41±8.13^b^ |  | *** | * |
| Met | 28.36±1.83^a^ | 32.00±1.72^a^ | 29.96±1.62^a^ | 18.22±1.95^b^ |  | *** | ** |
| Ile | 76.45± 5.74^a^ | 86.44±5.40^a^ | 76.97±5.06^a^ | 46.62±5.74^b^ |  | *** | * |
| Phe | 42.46±3.77^ab^ | 53.20±3.55^a^ | 47.53±3.33^a^ | 32.52±4.02^b^ |  | ** | * |
| Lys | 213.94±20.36^b^ | 284.15±17.98^a^ | 240.55±16.86^ab^ | 155.54±19.10^c^ |  | ** | * |
| His | 28.78±2.92^b^ | 42.37±2.77^a^ | 41.07±2.42^a^ | 29.68±2.74^b^ |  | NS | ** |
| Tau | 118.57± 1.00^a^ | 134.90±10.64^a^ | 119.78±1.00^a^ | 101.22± 1.00^ab^ |  | NS | NS |
| Ser | 156.66±10.47^a^ | 182.39±9.86^a^ | 166.39±9.24^a^ | 109.59±10.94^b^ |  | ** | ** |
| Gly | 170.86±14.27^ab^ | 202.50±13.43^a^ | 200.62±13.11^a^ | 133.85±14.27^b^ |  | * | ** |
| Ala | 260.85± 20.10^a^ | 304.78±18.92^a^ | 261.97±17.74^a^ | 163.19±20.10^b^ |  | *** | NS |
| Pro | 381.09 ±37.12^a^ | 426.78±30.74^a^ | 435.74±29.21^a^ | 275.23±31.79^b^ |  | ** | ** |
| Cit | 53.45± 3.60^a^ | 51.76 ±3.39^a^ | 50.62 ±3.18^a^ | 30.09± 3.60^b^ |  | *** | * |
| Tyr | 53.43± 3.81^a^ | 47.86±3.59^a^ | 47.72±3.36^a^ | 31.77±3.98^b^ |  | ** | NS |
| Orn | 34.29±2.64^a^ | 32.61±2.48^a^ | 32.54±2.33^a^ | 27.62±2.81^a^ |  | NS | NS |
| Asp | 22.01± 2.68^b^ | 32.02± 2.53^a^ | 31.21± 2.37^a^ | 19.15±2.97^b^ |  | NS | ** |
| Asn | 46.96±5.73^a^ | 59.44± 5.54^a^ | 56.88±5.06^a^ | 30.01±6.35^b^ |  | * | ** |
| Glu | 87.81± 4.89^b^ | 106.19±4.53^a^ | 91.91± 4.13^a^ | 73.28 ± 6.47^c^ |  | * | NS |
| Gln | 1753±108.61^b^ | 2137±102.25^a^ | 2194±95.87^a^ | 2155 ±150.06^a^ |  | * | NS |
| Leu | 98.34±7.61^a^ | 114.79±7.16^a^ | 98.37±6.72^a^ | 65.72±7.61^b^ |  | ** | * |
| Arg | 103.22±7.46^a^ | 122.83± 7.02^a^ | 109.86±6.58^a^ | 61.81±7.46^b^ |  | *** | ** |

^1^ Cited by Tong et al.(2016).

^2^*, *P*<0.05, **, *P*<0.01, ***, *P*<0.001, NS: not significant.
